# Hsa‐miR‐335 enhances cell migration and invasion in lung adenocarcinoma through targeting Copine‐1

**DOI:** 10.1002/mco2.93

**Published:** 2021-11-04

**Authors:** Yang Wang, Jing Zhang, Li‐Ye Zhong, Shang‐Jia Huang, Nan‐Nan Yu, Lan Ouyang, Yu‐Long Niu, Jun‐Xiong Chen, Chun‐Hua Lu, Qing‐Yu He

**Affiliations:** ^1^ MOE Key Laboratory of Tumor Molecular Biology and Key Laboratory of Functional Protein Research of Guangdong Higher Education Institutes, Institute of Life and Health Engineering, College of Life Science and Technology Jinan University Guangzhou China; ^2^ The First Affiliated Hospital Jinan University Guangzhou China; ^3^ Gastrointestinal Surgery The First People's Hospital of FoShan Foshan China; ^4^ Guangdong Provincial Key Laboratory of Colorectal and Pelvic Floor Diseases, Guangdong Institute of Gastroenterology, The Sixth Affiliated Hospital Sun Yat‐sen University Guangzhou Guangdong China; ^5^ Research Laboratory of Zhuang & Yao Medicine Guangxi International Zhuang Medicine Hospital Affiliated to Guangxi University of Chinese Medicine Nanning Guangxi China

**Keywords:** CPNE1, lung adenocarcinoma, metastasis, miR‐335

## Abstract

Lung adenocarcinoma (LAC) is one of the most common pulmonary adenocarcinomas with a high peak of mortality, and metastasis is the main culprit of LAC deaths. microRNAs play important role in cancer metastasis, and thus are regarded as potential diagnostic and prognostic markers for human cancers. However, many miRNAs exhibit dual roles in diverse cellular contexts. Here, we revealed that hsa‐miR‐335, a previously reported tumor suppressor, exhibited an oncogenic role in LAC. Overexpression of miR‐335 enhanced the abilities of A549 and H1299 cells to invade and migrate by regulating epithelial‐mesenchymal transition, while inhibition of miR‐335 exhibited an opposite effect in vitro and in vivo. Mechanically, miR‐335 inhibited the expression of Copine‐1 (CPNE1), an NF‐κB suppressor, through interacting with its mRNA 3′UTR, while mutating the binding sites abolished this inhibitory effect. This finding not only highlights the suppressive effect of CPNE1 on cell motility, but also provides new insight into miR‐335 in promoting LAC metastasis.

## Graphical abstract

Hsa‐miR‐335, a previously reported tumor suppressor in multiple cancers, exhibits an oncogenic role in lung adenocarcinoma. miR‐335 increases the invasion and migration of cancer cells by regulating epithelial‐mesenchymal transition, while inhibition of miR‐335 exhibits opposite effect in vitro and in vivo. Mechanically, miR‐335 inhibits the expression of Copine‐1 (CPNE1), an NF‐κB suppressor, through interacting with its mRNA 3′UTR, while mutating the binding sites abolished this inhibitory effect. This finding not only highlights the suppressive effect of CPNE1 on cell motility, but also provides new insight into miR‐335 in promoting lung adenocarcinoma metastasis.

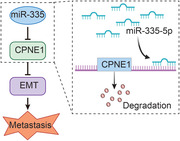

## 1 INTRODUCTION

Lung adenocarcinoma (LAC) is one of the most common malignant pulmonary adenocarcinomas with a high peak of mortality, which accounts for more than half of lung cancer,^1^ the 5‐year overall survival ratio still lower than 20%.^2^ Metastasis is the main culprit of LAC deaths and treatment failure for advanced LAC. Acquiring high motility is key for cancer dissemination. During this multistage progress, primary tumor cells undergo cell morphogenetic alteration, junction dissociation, and cytoskeleton rearrangement to acquire mesenchymal phenotype.^3^ Malignant behavior that transition from epithelial to mesenchymal (EMT) links to a wide range of advanced cancers that result in dismal clinical outcome.^4^

microRNAs (miRNAs) are a class of small non‐protein‐coding RNAs with 20–24 nucleotides involved in almost important biological processes. They exhibit their functions in post‐transcriptional regulation of gene expression via targeting to the 3′untranslated region (3′UTR) of the target mRNA for suppressing their expression. Accumulating evidence uncovered the key regulatory role of miRNAs in cancer metastasis,^5^, ^6^ and these metastasis‐associated miRNAs have high diagnostic and prognostic values for clinical application in human cancer.^7^ For example, miR‐21 is a well‐recognized onco‐miRNA that preventing a wide range of key tumor suppressor gene expression, such as phosphatase and tensin homolog (PTEN). In non‐small cell lung cancer (NSCLC), miR‐21 is upregulated in cancer tissue and therefore had been considered as a promising diagnostic and therapeutic marker in NSCLC.^8^ However, emerging studies revealed that many miRNAs exhibit dual roles in different cellular contexts.^9^, ^10^ Specially in cancer, some miRNAs are reported to exert contrary oncogenic or tumor‐suppressive functions, relying on whether their targets are potential tumor suppressors or oncogenes.^11^ For example, miR‐205, a highly conserved miRNA that exhibits both oncogenic and tumor suppressive role in various cancers,^12^ by which promotes lung cancer proliferation and metastasis by targeting SMAD4^13^ and PTEN,^14^ while reverses EMT progress in skin cancers by targeting zinc finger E‐box binding homeobox 1 (ZEB1) and ZEB2.^15^ Therefore, comprehensively analyzing the functionality of certain miRNAs is critical prior to their clinical applications.

miR‐335 is a critical microRNA with dual roles in cancer that attracted extensive attention. It was reported as a cancer suppressor in multiple cancers, such as prostate cancer, breast cancer and osteosarcoma,^16^, ^17^, ^18^ and even was considered as a negative diagnostic and prognostic marker. For example, miR‐335 suppresses cell proliferation and revascularization ability in prostate cancer via targeting early growth response 3 (Egr3).^17^ However, in gallbladder carcinoma, miR‐335 was reported to enhance cell proliferation by inhibiting myocyte enhancer factor 2D (MEF2D).^19^ Here, our study newly reported an oncogenetic role of miR‐335 in lung adenocarcinoma, by which miR‐335 enhances migratory and invasive abilities of lung cancer cells via targeting copine‐1 (CPNE1), an NF‐κB signaling suppressor. This finding provides another insight of miR‐335 in cancer metastasis.

## 2 RESULTS

### 2.1 miR‐335 overexpression increases cell migration and invasion

**FIGURE 1:**
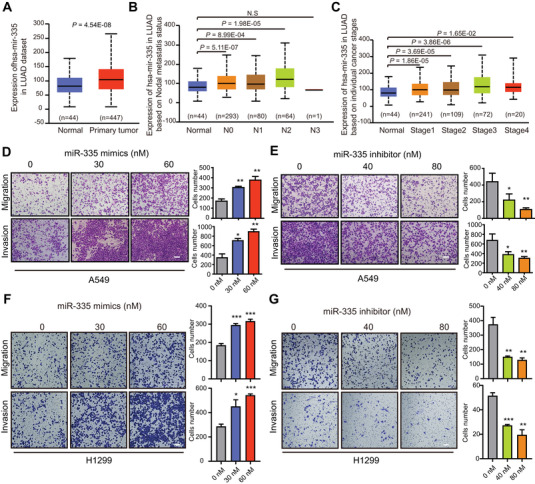
The clinical and functional relevance of miR‐335 in LAC metastasis. (A) Expression of miR‐335 was upregulated in TCGA LUAD tumors (n = 447), as compared to normal lung tissues (n = 44). (B) Among these patients, miR‐335 expression level was positively correlated with nodal metastasis status (N0‐N3), as well as (C) individual cancer stages (tumor samples classified to stage1, stage2, stage3, and stage4). (D‐G) miR‐335 promotes LAC cell migration and invasion. A549 (D‐E) and H1299 cells (F‐G) were transfected with miR‐335 (0, 30, 60 nM) or miR‐335 inhibitor (0, 40, 80 nM) for 24 h, and then subjected to transwell assays for comparing their migration and invasion abilities. Bars, SEM; *P < 0.05; **P < 0.01; ***P < 0.001. Scale bar, 100 μm

**FIGURE 2:**
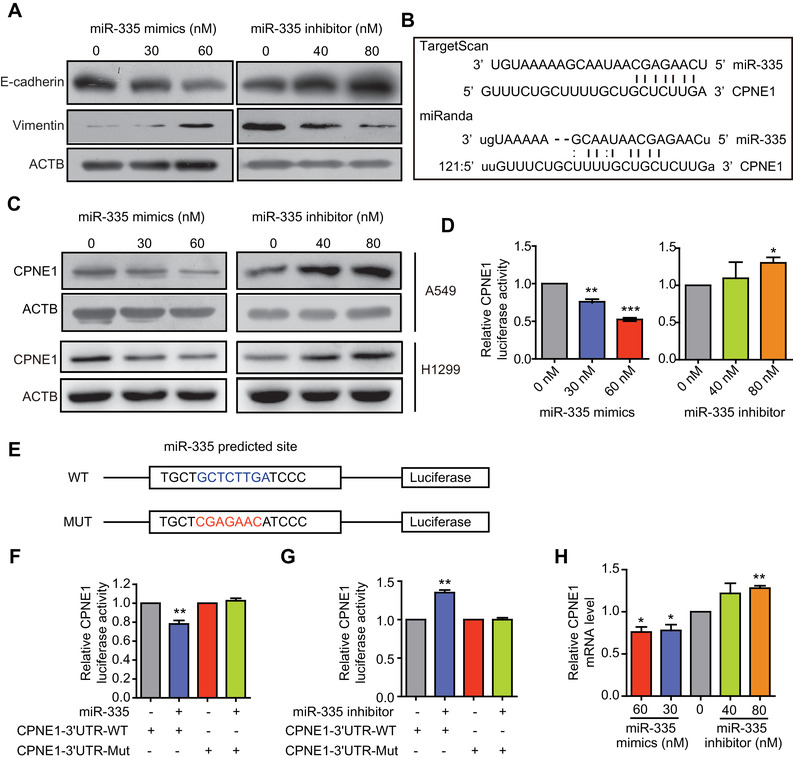
miR‐335 targets 3′UTR of CPNE1. (A) miR‐335 promotes epithelial‐to‐mesenchymal transition of lung cancer cell. A549 cells were transfected with miR‐335 mimics (0, 30, 60 nM) for 24 h, and then the cell lysates were subjected to Western blotting for determining the expression of vimentin and E‐cadherin. (B) The binding site of miR‐335 in the 3′UTR of CPNE1 was simultaneously predicted by the TargetScan and miRanda software; (C) The expression of CPNE1 in A549 and H1299 cells transfected with increasing concentrations of miR‐335 mimics or inhibitor, was determined by Western blotting. (D) Luciferase reporter assays determined the binding between miR‐335 and 3′UTR of CPNE1. A549 cells were transfected with constructed luciferase reporter containing predicted binding sequence and the elevating concentrations of the miR‐335 mimics or inhibitor, the values of luciferase activity were measured. (E) Schematic diagram showing the miR‐335 binding site in the 3′UTR region of the CPNE1 gene. (F and G) A549 cells were co‐transfected with the luciferase reporter with or without mutation of binding sites and the indicated concentrations of the miR‐335 mimics or inhibitor, the values of luciferase activity were measured. (H) qRT‐PCR assay detecting the mRNA level of CPNE1 in A549 cells transfected with increasing concentrations of miR‐335 mimics or inhibitors. Bars, SEM; *P < 0.05; **P < 0.01

Firstly, to determine the clinical significance of miR‐335 in LAC, TCGA database was performed for analysis, and found that miR‐335 was upregulated in lung adenocarcinoma (LUAD) tissue (*P* = 4.54 × 10^‐08^; Figure 1A). Moreover, the expression of miR‐335 was not only positively relevant to N0, N1 and N2 stage of LUAD patients (Figure 1B), but also upregulated along with increasing stage (Figure 1C). We therefore speculated that miR‐335 plays an oncogenic role in LAC development. To examine the effect of miR‐335 on cell mobility, both A549 and H1299 cells were transfected with miR‐335 mimics (up to 60 nM) or the corresponding inhibitor (up to 80 nM), respectively, and the cell migratory and invasive abilities were then determined by transwell assay (Figure 1D–G). We found that miR‐335 mimics enhanced the cell migration and invasion (Figure 1D,F), while suppressed miR‐335 expression had an opposite effect (Figure 1E,G). Correspondingly, we observed that miR‐335 mimics increased the expression of mesenchymal marker vimentin but decreased the epithelial marker E‐cadherin, while treatment with miR‐335 inhibitor showed opposite effect (Figure 2A). These results suggest that miR‐335 positively regulated epithelial‐mesenchymal transition to enhance the migration and invasion of LAC.

### 2.2 miR‐335 binds to the 3′UTR of CPNE1 mRNA

To investigate the mechanism for how miR‐335 promotes cell mobility, two predictive tools including TargetScan (http://www.targetscan.org/) and miRanda were performed to analyze the potential targets of miR‐335, as shown in Figure 2B, the result showed that miR‐335 likely bind to the 3′ UTR of CPNE1 mRNA. To validate this prediction, we performed Western blotting to detect the protein expression of CPNE1 in both LAC cells treated with miR‐335 mimics or inhibitors. The expression of CPNE1 was negatively correlated with miR‐335 (Figure 2C), suggesting that miR‐335 promotes LAC cell migration and invasion by inhibiting CPNE1. To verify the binding of miR‐335 on CPNE1 3′UTR, the predicted matching sequence was inserted into dual‐luciferase reporter, then the overexpression of miR‐335 was found to decrease the luciferase activity, while miR‐335 suppression enhanced the luciferase activity (Figure 2D). Moreover, the 3′UTR of CPNE1 containing the miR‐335 matching sequence, and CPNE1 MUT 3′UTR with miR‐335 mutated binding sequence were constructed (Figure 2E), respectively, for dual‐luciferase reporter assay in A549 cells. The data showed that luciferase activity was not changed by either over‐expression or inhibition of miR‐335 when the CPNE1 3′UTR sequences in the complementary sites for the seed region of miR‐335 were mutated (Figure 2F,G), suggesting that miR‐335 directly targets 3′ UTR of CPNE1 mRNA. In addition, qRT‐PCR revealed that miR‐335 could decrease the mRNA level of CPNE1, while miR‐335 inhibitor increased the mRNA level of CPNE1 (Figure 2H), confirming that miR‐335 binds to CPNE1‐3′UTR for degrading the mRNA.

### 2.3 CPNE1 suppresses cell migration and invasion in LAC

**FIGURE 3:**
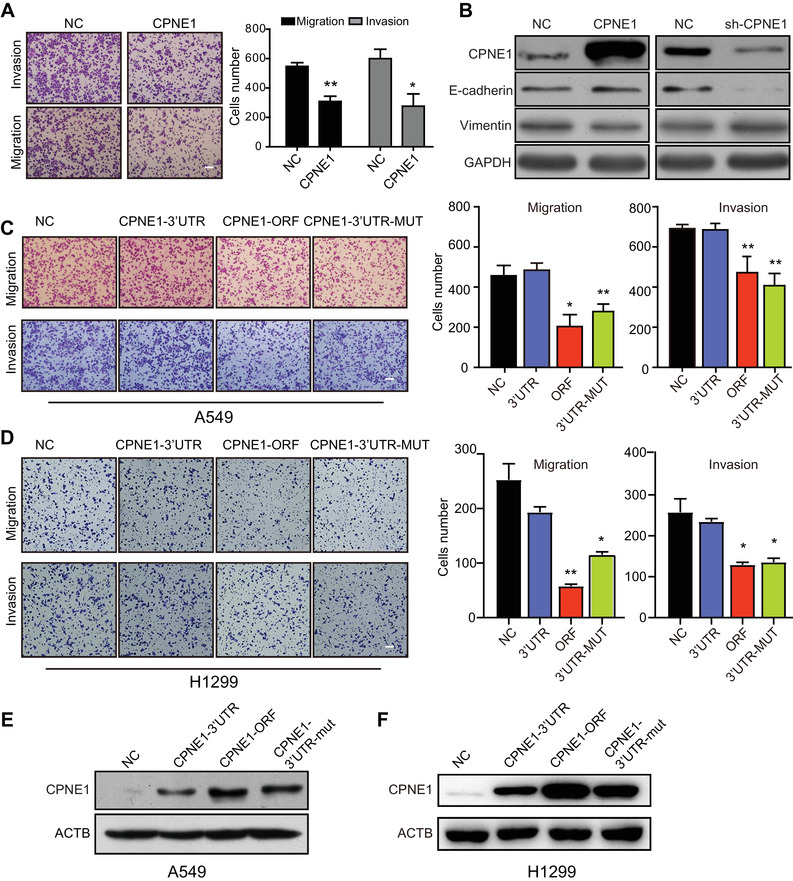
Inhibitory role of CPNE1 in cell migration and invasion of LAC. (A) A549 cells with or without overexpressing CPNE1 were compared for their migration and invasion abilities by transwell assays. (B) The expressions of vimentin and E‐cadherin in A549 with CPNE1 overexpression or knockdown were detected by Western blotting. (C‐F) A549 and H1299 cells were transfected with the indicated plasmids, and the migration and invasion abilities (C, D) and CPNE1 protein level (E, F) were determined by transwell assay and Western blotting, respectively. Bars, SEM; *P < 0.05; **P < 0.01. Scale bar, 100 μm

We next sought to determine the role of CPEN1 in cell mobility. As shown in Figure 3A, CPNE1 overexpression markedly suppressed the abilities of A549 cells to migrate and invade. Since EMT is a critical progress that is involved in the initiation or maintenance of cancer metastasis in multiple malignancies,^20^ we further examined the regulatory role of CPNE1 in EMT process. Western blotting assay showed that an increase of E‐cadherin and a decrease of vimentin in cells with CPNE1 overexpression, whereas opposite effects were observed in CPNE1‐knockdown cells (Figure 3B). Collectively, these results suggested that CPNE1 is a tumor suppressor in regulating EMT progression, preventing the migration and invasion of LAC cells.

**FIGURE 4:**
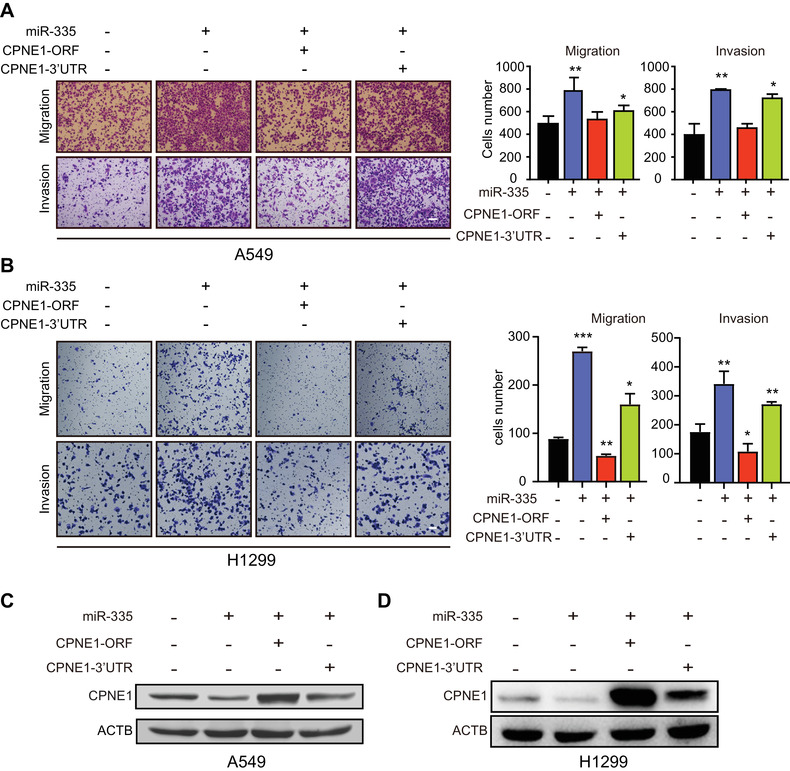
miR‐335 targets 3′UTR of CPNE1 for promotion of cell migration and invasion. (A‐D) A549 and H1299 cells were co‐transfected with CPNE1‐ORF, CPNE1‐3′UTR or CPNE1‐3′UTR‐mutation plasmids and the miR‐335, for comparing their migration, invasion abilities (A‐B) and CPNE1 protein level (C‐D) by using transwell assay and Western blotting, respectively. Bars, SEM; *P < 0.05, **P < 0.01; ***P < 0.001. Scale bar, 100 μm

We further asked whether CPNE1 is critical for miR‐335‐mediated cell mobility. CPNE1‐ORF (open reading frame), CPNE1‐3′UTR and CPNE1‐3′UTR mutation plasmids were respectively transfected into LAC cells to compare their effects on cell mobility. Results from transwell assay showed that the expression of CPNE1‐ORF and the CPNE1‐3′UTR mutant, but not the CPNE1‐3′UTR, abolished the migratory and invasive abilities of A549 and H1299 cells (Figure 3C,D). The protein level of CPNE1 was increased in the cells transfected with CPNE1‐ORF and CPNE1‐3′UTR mutant, since CPNE1 mRNA with the absence of miR‐335 binding sites was resistant to miR‐335. In contrast, the expression of CPNE1 was suppressed by endogenous miR‐335 due to the existence of the 3′UTR (Figure 3E,F). Moreover, both A549 and H1299 cells expressing miR‐335 transfected with CPNE1‐ORF, but not CPNE1‐3′UTR, could restore the migration and invasion ability enhanced by miR‐335 (Figure 4A,B). The same treatment was subjected to Western blotting analysis, showing that CPNE1 was decreased by miR‐335, while only CPNE1‐ORF, rather than CPNE1‐3′UTR could rescue its expression (Figure 4C,D).

### 2.4 Inhibition of miR‐335 decreases cancer metastasis in vivo

**FIGURE 5:**
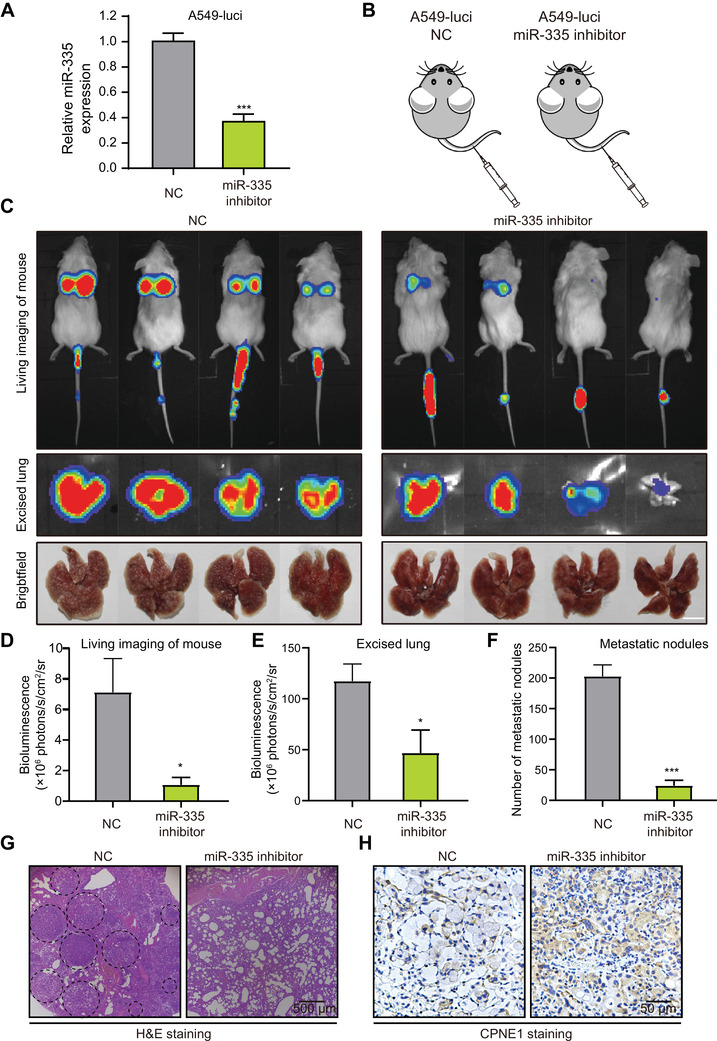
miR‐335 inhibitor suppresses cancer metastasis in vivo. (A) The establishment of miR‐335 stabling knockdown cell line, qRT‐PCR assay detected the expression level of miR‐335 in A549‐luci cells stabling expressing miR‐335 inhibitor. (B) Experimental scheme illustrating the animal experiment design. (C‐D) Bioluminescence imaging and quantification of lung metastasis in the NCG mice that were intravenously injected with A549‐luci cells expressing miR‐335 inhibitor or NC. The lungs were excised for bioluminescence imaging and quantification (E), and the metastatic nodules in lungs were quantified (F). Scale bar, 0.5 cm. The pulmonary metastases in the mouse model were histologically analyzed by H&E staining (G) and CPNE1 staining (H). Bars, SD; *P < 0.05; ***P < 0.001. Dashed circle, metastatic nodules.

We next confirmed the pro‐metastatic effect of miR‐335 in a mouse model, luciferase‐labeled A549 cell line with stabling expressed miR‐335 inhibitor (Figure 5A) was established for metastatic mouse model (Figure 5B). The result showed that luciferase‐labelled A549 cells with stabe expression of miR‐335 inhibitor showed less metastasized to lung surface than control group, as revealed by living imaging. (Figure 5C–E). More lung metastatic nodules were formed in the lungs of the NC group (Figure 5F), as confirmed by histological analysis including H&E staining (Figure 5G) and CPNE1 staining (Figure 5H). Taken together, our data demonstrated that suppression of miR‐335 decreases lung cancer metastasis in vivo.

## 3 DISCUSSION

As a main culprit of LAC deaths, metastasis links to poor prognosis in patients, therefore, a deeper understanding of the potential mechanism of LAC metastasis would provide an effective strategy for inhibiting LAC dissemination, along with identifying new regulators of the multi‐step progression. Here, we reported a novel molecular function of miR‐335 and CPNE1 in regulating cancer metastasis, by which increment of miR‐335 induced cell migration and invasion through binding to the 3′UTR of CPNE1 for inhibiting its mRNA stability.

In previous studies, the dual role of miR‐335 was found in multiple cancers. For example, it increases the cell sensitivity toward cisplatin in ovarian cancer through silencing BCL2 linke 2 (BCL2L2).^21^ Given its high abundance in extracellular vesicles, exosomal miR‐335 was adopted as a therapeutic strategy for hepatocellular cancer therapy.^22^ However, recent study showed that exosomal miR‐335‐5p increases colorectal cancer metastasis via reducing RAS p21 protein activator 1 (RASA1).^23^ Interestingly, the dual roles of miR‐335 was even reported in the same cancer type. For instance, in gastric cancer, a high level of miR‐335 was associated with a high frequency of recurrence and poor survival.^24^ However, miR‐335 was reported to targeted rho associated coiled‐coil containing protein kinase 1 (ROCK1) that resulted in a decrease in cell proliferation and invasion in BGC‐823 cell line.^25^ Though tumor‐suppressive role of miR‐335 in lung cancer was reported,^26^ here, we found that miR‐335 promoted cell invasion and metastasis of LAC cells in vitro and in vivo. This finding provides experimental evidence for the dual roles of miR‐335 in cancers.

CPNE1, a calcium phospholipid‐binding protein, was previously reported as a NF‐κB repressor, which physically interacts with p65 and induces endoproteolysis of p65 to suppress NF‐κB signaling activation.^27^ NF‐κB is a dynamic transcription factor that regulates many important biological processes (such as EMT) involved in cancer metastasis.^28^ In this study, we found that overexpression of CPNE1 decreased cell migration and invasion, it is therefore rational to speculate that CPEN1 blocks cell EMT via inhibiting NF‐κB signaling. Though recent studies revealed that overexpression of CPNE1 improved tumor proliferation and metastasis, associated with poor prognosis and malignancy.^29^, ^30^ Our work demonstrated that CPNE1 expression handicapped cell motility by decreasing EMT progress, while the motility‐suppressive effect could be abolished by miR‐335 treatment. The inhibitory effect of miRNAs on gene expression is usually based on hindering target mRNA protein translation or recruiting endonuclease for mRNA degradation.^31^ In this study, our qRT‐PCR assay revealed that miR‐335 could decrease the mRNA level of CPNE1, indicating that miR‐335 binds to 3′UTR of CPNE1 mRNA for degradation.

To further confirm the role of miR‐335 and CPNE1 in LAC invasion, we overexpressed CPNE1‐ORF and the ORF containing‐3′UTR with or without mutant, and found that mRNA of CPNE1 bearing 3′UTR can be targeted by endogenous miR‐335, showing no significant change in cell migration and invasion. Expression of CPNE1‐ORF and ORF‐3′UTR‐MUT protected their degradation from endogenous miR‐335, showing strong inhibitory effect on cell migration and invasion. Although the role of miR‐335 and CPNE1 we revealed in lung cancer was contrary to previous findings, it is interesting to ask whether the molecular function of miR‐335 and CPNE1 depends on any specific condition. The functional divergence presented by miR‐335 and CPNE1 in cancers implicates the complexity and heterogeneity rendered by various tumors.

In conclusion, we here revealed that miR‐335 promoted cell EMT, migration, and invasion through binding to the 3′UTR of CPNE1 for degrading its mRNA. We also characterized the suppressive role of CPNE1 in cell motility, highlighting a new molecular function and cancer phenotype regulated by miR‐335 and CPNE1 in lung cancer.

## 4 MATERIALS AND METHODS

### 4.1 Culture conditions of cell lines

Human lung adenocarcinoma cell lines A549 and H1299 cells were purchased from American Type Culture Collections (ATCC, Rockville, MD). Cells were cultured with DMEM medium (Thermo Fisher Scientific, Waltham, MA, USA) with 10% fetal bovine serum (FBS, Gibco‐Invitrogen Corporation, CA), 1% penicillin/streptomycin (GBCBIO Technologies, Guangzhou, China), and 10 mg/mL ciprofloxacin under standard cell maintain conditions of 5% CO_2_.

### 4.2 Plasmids, transfection, and infection

The CPNE1‐overexpression was obtained by PCR amplification (forward, 5′‐ GCGGGAATTCGGTAATTCGGGGTCTGGGTTCT‐3′ and reverse, 5′‐GGCGTCTA GATCTGGGACACAGGATTGAGGA‐3′) cloned into the pcDNA3.1 vector. And the CPNE1‐knockdown plasmid expressing shRNA sequences (5′‐CACACAACTGG TCTCATACTT‐3′) were generated in the pLKO.1 vector. The 3′UTR‐WT and 3′UTR‐mut of CPNE1 were introduced into psiCHECK2 plasmid. The primers for CPNE1‐3′UTR‐WT forward, 5′‐GGACGAATTCGCGGGGGGTAATTCGGGGT‐3′ and reverse, 5′‐CGACTCTAG ATGTCAGGAGCAAAGGTGG‐3′. The primers for CPNE1‐3′UTR‐mut forward, 5′‐ATACTTGTTTCTGCTTTTGCTGGAGAAGATCC CACCTTTGCTCCTG‐3′ and reverse, 5′‐CAGGAGCAAAGGTGGGATCTTCTCCA GCAAAAGCAGAAACAAGTAT‐3′. The miR‐335 mimic (UGUUUUGAGCGGGG GUCAAGAGCAAUAACGAAAAAUGUUUGUCAUAAACCGUUUUUCAUUAUUGCUCCUGACCUCCUCUCAUUUGCUAUAUUCA), miR‐335 inhibitor (CATTT TTCGTTATTGCTCTTG) and their negative control were obtained from Ambion (Austin, TX, USA). Lipofectamine 3000 reagent obtained from Thermo Fisher Scientific used for cell transfection according to the manufacturer's instruction.

### 4.3 Migration and invasion assays

Cell migration ability was determined by using the uncoated Transwell chambers (8 μm pore size; BD Biosciences) as we described previously.^21^ Cells of 3 × 10^5^ in serum‐free medium were seeded to the upper chamber, and the complete medium was added to the bottom chamber. After 24 h, the cells that had migrated to the lower surface were fixed with methanol and stained with 0.2% crystal violet. Images of four random fields were captured for counting the number of migrated cells. For the invasion assay, 100 μL of Matrigel (Corning Incorporated, Corning, NY, USA) were added to each chamber and placed at 37°C for 15 min, then similar protocol to migration assay was carried out.

### 4.4 Western blotting and IHC staining

Proteins were extracted by cell lysis buffer (Cell Signaling Technology, Danvers, MA, USA) with PMSF and quantified by BCA assay (Thermo Fisher Scientific). The proteins were separated on 10% or 12% SDS‐PAGE and then electrotransferred to a PVDF transfer membranes (Millipore, Bedford, MA, USA). Primary antibodies for E‐cadherin (Cell Signaling Technology), vimentin (Cell Signaling Technology), CPNE1 (Proteintech, Wuhan, China), β‐actin (Proteintech), GAPDH (Proteintech), and the secondary antibodies (Proteintech) were used for immunoblotting. Immunohistochemistry analysis was performed as previously described.^32^ The antibody of anti‐CPNE1 for IHC was obtained from Abcam (Cambridge, UK). The stained sections were imaged using fluorescence microscopy (Nikon Eclipse C1).

### 4.5 Bioinformatics study

The expression of miR‐335 in LAC was analyzed by using GEPIA (Gene Expression Profiling Interactive Analysis) webserver (http://gepia.cancer‐pku.cn/). To identify novel targets of miR‐335, two software programs including TargetScanHuman (http://www.targetscan.org/) and miRanda (http://www.microrna.org/microrna/getExpr) were used to predict the biological targets of miR‐335.

### 4.6 Luciferase reporter assay

Cells were co‐transfected with psiCHECK2‐CPNE1‐3′UTR plasmid and miR‐335 or miR‐335 inhibitor for 48 h. The luciferase activity was measured by using dual‐luciferase reporter assay (Promega, WI, USA) according to the manufacturer's instructions.

### 4.7 Animal studies

Female NCG mice (aged 4–5 weeks), were purchased from GemPharmatech Co., Ltd. (Nanjing, Jiangsu, China) and maintained under defined conditions at the Institute of Laboratory Animal Science at Jinan University (SPF grade). For in vivo metastatic assays, 1 × 10^6^ luciferase‐tagged A549 cells with stably expressing of miR‐335 inhibitor, and the corresponding negative control cells were injected into the tail veins of NCG mice. Five weeks after injection, the metastatic foci in the lungs were visualized using the IVIS 200 Imaging System (Xenogen, CA, USA). All animal experiments were approved by the Laboratory Animal Ethics Committee of Jinan University and conformed to the US Department of Health and Human Services Guide for the Care and Use of Laboratory Animals

### 4.8 Quantitative RT‐PCR

Total RNA was extracted using trizol reagent (Thermo Fisher Scientific) according to the manufacturer's protocol. cDNA was synthesized using the PrimeScriptTMRT reagent Kit (Takara Biomedical Technology, Beijing, China), and qPCR was performed using the miScript SYBR® Green PCR Kit (Takara) and Applied Biosystems StepOne Real‐Time PCR System (Thermo Fisher Scientific). The cDNA was amplified by PCR using the following specific primers: CPNE1 forward, 5′‐CACTGCGTGACCTTGGTTCA‐3′ and reverse, 5′‐ TCCCACATCCTGTAAAAG GAC‐3′. GAPDH forward, 5′‐GAAGGTGAAGGTCGGAGTC‐3′ and reverse, 5′‐TGGGATCCTCTAGCTGTGGATAGTG‐3′. The expression of miR‐335‐5p was detected by using Hairpin‐itTM Real‐Time PCR Kit obtained from GenePharma, (Shanghai, China) with the following specific primers: miR‐335‐5p forward: 5′‐ CAGAATAGTCAAGAGCAATAACG‐3′, miR‐335‐5p reverse: 5′‐ TATGGTTGTTCACGACTCCTTCAC‐3′. U6 snRNA‐forward: 5′‐ CGCTTCGGCAGCACATATAC‐3′, U6 snRNA‐reverse: 5′‐ TTCACGAATTTGCGTGTCATC‐3′.

### 4.9 Statistical analysis

One‐way ANOVA and two‐tailed Student's tests were performed using GraphPad Prism software v.5.01. The values were presented as the mean ± SEM from three independent experiments. The differences with *P < 0.05; **P < 0.01; ***P < 0.001 were considered statistically significant.

## CONFLICT OF INTEREST

The authors declare that they have no conflict of interest.

## ETHICS APPROVAL

The animal experiments were approved by the Animal Experimental Ethics Committee of Jinan University.

## AUTHOR CONTRIBUTIONS

Qing‐Yu He and Yang Wang conceived and designed the project. Yang Wang, Jing Zhang, and Li‐Ye Zhong performed the experiments. Shang‐Jia Huang, Nan‐Nan Yu, Lan Ouyang, Yu‐Long Niu, Jun‐Xiong Chen, and Chun‐Hua Lu participated in the scientific discussion and research design. Yang Wang wrote and revised the manuscript.

## Data Availability

All data are available from the corresponding authors upon request.
